# Impaired cerebral autoregulation is associated with brain dysfunction in patients with sepsis

**DOI:** 10.1186/s13054-018-2258-8

**Published:** 2018-12-04

**Authors:** Ilaria Alice Crippa, Carles Subirà, Jean-Louis Vincent, Rafael Fernandez Fernandez, Silvia Cano Hernandez, Federica Zama Cavicchi, Jacques Creteur, Fabio Silvio Taccone

**Affiliations:** 1 Department of Intensive Care, Erasme Hospital, Université Libre de Bruxelles (ULB), Route de Lennik, 808, 1070 Brussels, Belgium; 2 Department of Intensive Care, Althaia Xarxa Assistencial Universitària de Manresa, Barcelona, Spain; 3 CIBERES, Madrid, Spain

**Keywords:** Doppler sonography, Transcranial, Cerebrovascular circulation, Cerebral blood flow, Brain dysfunction

## Abstract

**Background:**

Sepsis-associated brain dysfunction (SABD) is associated with high morbidity and mortality. The pathophysiology of SABD is multifactorial. One hypothesis is that impaired cerebral autoregulation (CAR) may result in brain hypoperfusion and neuronal damage leading to SABD.

**Methods:**

We studied 100 adult patients with sepsis (July 2012–March 2017) (age = 62 [52–71] years; Acute Physiology and Chronic Health Evaluation II score on admission = 21 [15–26]). Exclusion criteria were acute or chronic intracranial disease, arrhythmias, extracorporeal membrane oxygenation, and known intra- or extracranial supra-aortic vessel disease. The site of infection was predominantly abdominal (46%) or pulmonary (28%). Transcranial Doppler was performed, insonating the left middle cerebral artery with a 2-MHz probe. Middle cerebral artery blood flow velocity (FV) and arterial blood pressure (ABP) signals were recorded simultaneously; Pearson’s correlation coefficient (mean flow index [Mxa]) between ABP and FV was calculated using MATLAB. Impaired CAR was defined as Mxa > 0.3.

**Results:**

Mxa was 0.29 [0.05–0.62]. CAR was impaired in 50 patients (50%). In a multiple linear regression analysis, low mean arterial pressure, history of chronic kidney disease and fungal infection were associated with high Mxa. SABD was diagnosed in 57 patients (57%). In a multivariable analysis, altered cerebral autoregulation, mechanical ventilation and history of vascular disease were independent predictors of SABD.

**Conclusions:**

Cerebral autoregulation was altered in half of the patients with sepsis and was associated with the development of SABD. These findings support the concept that cerebral hypoxia could contribute to the development of SABD.

**Electronic supplementary material:**

The online version of this article (10.1186/s13054-018-2258-8) contains supplementary material, which is available to authorized users.

## Introduction

Sepsis is a life-threatening clinical condition associated with severe infection [1]. Sepsis-associated brain dysfunction (SABD) is considered as cerebral dysfunction that accompanies sepsis in the absence of central nervous system infection and other possible causes of brain dysfunction (i.e., structural central nervous system lesions or drug overdose) [2]. SABD is probably the most frequent sepsis-related organ dysfunction, affecting up to 70% of patients with sepsis and frequently occurring early, often before any other organ involvement [3]. The development of SABD has been associated with higher mortality, lower quality of life in survivors and long-term neurological sequelae [4]. Unfortunately, owing to the unspecific spectrum of symptoms, SABD remains a diagnosis of exclusion, usually contextualized in daily clinical practice according to the patient’s history.

The pathophysiology of SABD is still unclear and probably multifactorial, involving diffuse neuroinflammation, excitotoxicity and cerebral ischaemia [5]. In the absence of vascular occlusion, cerebral ischaemia in patients with sepsis may be the result of reduced cerebral blood flow (CBF) secondary to hypotension [6, 7]. Moreover, alterations in cerebral microcirculation may occur even with normal systemic haemodynamics and could contribute to tissue hypoxia [8, 9]. As such, an alteration in cerebral autoregulation (CAR) (i.e., the homeostatic mechanism that protects the brain tissue from the potentially damaging effects of hypo- and hyperperfusion) could be considered as the main determinant of cerebral ischaemia during sepsis because small cerebral arterioles, which are an essential part of the microcirculation and of CAR, could become unable to actively regulate their calibre and maintain CBF constant over a wide range of external stimuli, including cerebral perfusion pressure (CPP) or carbon dioxide [10]. Some authors have suggested that CAR may be altered in patients with sepsis. However, previous studies involved small cohorts of patients, used different CAR assessment methods and did not consider some potential confounders, such as severity of disease, use of vasopressors, age or extracerebral organ dysfunction, in the association of impaired CAR with brain dysfunction [11–15]. The aim of this study was therefore to evaluate the association of altered CAR with the occurrence of SABD, as well as to identify clinical factors associated with altered CAR in patients with sepsis.

## Methods

### Study design and population

This prospective, observational study was conducted between June 2012 and March 2017 in the intensive care units (ICUs) of Althaia Foundation Hospital (from June 2012 to June 2015; University of Manresa, Barcelona, Spain) and Erasme Hospital (from January 2015 to February 2017; Université Libre de Bruxelles, Belgium). All patients treated for sepsis were considered for inclusion. Sepsis was defined using standard criteria [16]. Exclusion criteria were age < 18 years, previous chronic or acute intracranial disease, known intra- or extracranial vascular stenosis, presence of arrhythmias or a pacemaker, mechanical cardiac support (i.e., veno-arterial extracorporeal membrane oxygenation, left ventricular assist device, intra-aortic balloon pump counterpulsation), severe hypotension (mean arterial pressure [MAP] < 50 mmHg), severe hypercapnia (arterial carbon dioxide partial pressure [PaCO_2_] > 65 mmHg), pregnancy, end-of-life care, death or ICU discharge within 24 h from admission, sepsis or septic shock diagnosed more than 48 h prior to study inclusion, transtemporal bone window inadequate for accurate transcranial Doppler (TCD) examination, absence of invasive arterial blood pressure (ABP) monitoring, and consent refusal. Because of the observational nature of the study, patients were only included during working hours and when the TCD operator (IAC or CS) was available. Patients were treated according to local guidelines/routine clinical practice. The study protocol was approved by local ethics committees, and informed consent was obtained from the patient or her/his legal representative.

### Data collection

We collected demographic data, pre-existing comorbid diseases and the Acute Physiology and Chronic Health Evaluation II (APACHE II) score on admission. We recorded the site of infection, the pathogen(s) involved and the outcome at ICU discharge. Haemoglobin and C-reactive protein concentrations, as well as arterial oxygen partial pressure, PaCO_2_, pH, lactate concentration and central venous oxygen saturation [ScvO_2_], on the day of CAR assessment were noted. Use of sedation and/or neuromuscular blocking agents (NMBAs), dose of vasoactive medications (norepinephrine and dobutamine), ventilator setting and body temperature at the time of examination were also recorded. Given the clinically oriented, pragmatic nature of the study, SABD was defined as a Glasgow Coma Scale score < 15 or when disorientation, altered thinking or agitation was reported by the attending physician, independently of the use of sedatives/analgesics and in the absence of previous neurological diseases (i.e., dementia, cerebrovascular disease, brain tumours, previous traumatic brain injury). Neurological assessment of these patients was routinely performed at least three times per day in both participating centres over the entire ICU stay. Clinicians were blinded to the CAR assessments. For the purpose of the analysis, patients in whom neurological evaluation was not possible because of continuous sedation (*n* = 6) were considered as having SABD.

### Transcranial Doppler and cerebral autoregulation assessment

We assessed CAR using the mean flow index (Mxa), a time domain index of dynamic CAR which uses ABP as a surrogate for CPP and modifications in blood flow velocity in intracranial vessels (FV) as a surrogate of modifications in CBF. ABP was recorded through invasive monitoring (in either the radial or femoral artery), and FV was assessed by TCD. TCD was performed once within 48 h of sepsis diagnosis using a 2-MHz monitoring probe (Compumedics DWL, Dresden, Germany) placed on the left transtemporal window to insonate the middle cerebral artery (MCA) and kept in place for at least 10 min using a special fixing device to ensure a constant angle of insonation. The MCA was identified as suggested in the literature [17]. ABP and FV were recorded simultaneously (Doppler-Box X; Compumedics DWL) and sampled at a frequency of 50 Hz. Signals were averaged on 10-s consecutive windows with 50% overlap for the entire length of the recording, then the Pearson’s correlation coefficient between the averaged ABP and flow velocity was calculated using MATLAB (MathWorks, Natick, MA, USA). Mxa is a continuous index, ranging from − 1 to + 1: values close to + 1 indicate a close positive relationship between MAP and FV, suggesting passive dependency of CBF and thus impaired CAR, whereas values around zero or negative values indicate independence of FV from MAP and thus intact CAR [18]. We defined altered CAR if Mxa was > 0.3 and intact CAR if Mxa was ≤ 0.3. Patients were maintained in steady-state conditions throughout the examination. No changes in respiratory conditions or in pharmacological or fluid therapy were allowed either in the 20 min before or during the TCD examination. Samples were examined automatically (by a custom-written script) and manually for artefacts; if artefacts were present, the entire cardiac cycle was discarded; if artefacts were > 10% of the total recording, the entire recording was discarded.

### Statistical analysis

Statistical analysis was performed using IBM SPSS® Statistics for Windows version 24 software (IBM, Armonk, NY, USA). Continuous variables are expressed as mean ± SD or median [25th–75th percentiles]. Categorical variables are expressed as count (percent). Distribution of continuous variables was tested by Shapiro-Wilk test and inspection of Q-Q plots. Student’s *t* test, Welch’s *t* test, or Fisher’s exact test was applied for comparisons between groups, as appropriate. Correlation was assessed using Pearson’s *r* or Spearman’s *r*_s_ coefficients. Binary logistic regression analyses were performed with SABD as the dependent variable; variables associated with the development of SABD during the ICU stay (*p* <  0.1) on a univariate basis were introduced in the multivariable model. A standard linear regression analysis to investigate the predictive value of pre-determined clinical and laboratory variables on Mxa was also conducted. Variables associated with Mxa (*p* <  0.1) on a univariate basis were introduced in the multivariable models. ROC analysis was used to evaluate the accuracy of Mxa to discriminate the occurrence of SABD in the study cohort. Youden’s index was computed to identify the Mxa with the best sensitivity and specificity to predict SABD. Outliers were assessed by standardized and studentized deleted residuals and box plot inspection. The Durbin-Watson statistic was used to test for autocorrelation in the residuals from the statistical regression model. Multi-collinearity was assessed using the variance inflation factor. Linearity of relationships was assessed using partial regression plot analysis and a Box-Tidwell procedure with Bonferroni correction as appropriate. Homoscedasticity was assessed by visual inspection of a plot of studentized residuals versus unstandardized predicted values. All analyses were adjusted for centre of enrolment. Statistical significance was set at *p* < 0.05 (two-sided).

## Results

### Study population

After exclusion of 6 patients because of artefacts, we analysed 100 patients (72 from the Erasme Hospital cohort and 28 from the Althaia Foundation Hospital cohort). The ICU length of stay (LOS) was 7 [4–13] days. The source of sepsis was most commonly abdominal (46%) or respiratory (28%). Gram-negative bacteria and gram-positive bacteria were involved in 45% and 35% of cases, respectively. At the time of the TCD evaluation, 48 patients were sedated with continuous infusion of one or more medications (i.e., 31 patients were receiving midazolam, 14 patients were receiving propofol and 19 patients were receiving opiates); 14 patients were receiving NMBAs; and 61 were receiving mechanical ventilation. Seventy-four patients were treated with norepinephrine (dose 0.17 [0–0.75] μg/min) and 16 with dobutamine (dose 3 [2.5–5.0] μg/kg/min). Heart rate (HR) was 96 [80–108] beats/min, and MAP was 76 [67–86] mmHg. No episodes of severe dysglycaemia or hypernatremia were reported in our study population. Characteristics of the study population are shown in Table 1. Characteristics in the two cohorts (Erasme Hospital and Althaia Foundation Hospital) are shown in Additional file 1: Table S3.

**Table 1:** Characteristics of the study population according to the occurrence of sepsis-associated brain dysfunction

	All (*N* = 100)	SABD (*n* = 57)	Non-SABD (*n* = 43)	*p* Value
Age, years	63 [52–72]	66 [52–75]	61 [53–68]	0.14
Male sex, *n* (%)	72 (72)	40 (70)	36 (84)	0.16
APACHE II score on admission	21 [15–26]	25 [18–29]	18 [13–24]	< 0.01
ICU LOS, days	7 [4–13]	9 [16–5]	6 [4–10]	0.03
Alive at ICU discharge, *n* (%)	76 (76)	36 (63)	40 (93)	< 0.01
Comorbidities				
CKD, *n* (%)	10 (10)	7 (12)	3 (7)	0.51
Vascular disease, *n* (%)	20 (20)	17 (30)	3 (7)	< 0.01
Diabetes mellitus, *n* (%)	23 (23)	12 (21)	10 (23)	0.81
Smoking, *n* (%)	19 (19)	8 (14)	11 (26)	0.20
Arterial hypertension, *n* (%)	45 (45)	23 (40)	21 (49)	0.42
At time of cerebral autoregulation assessment				
Sedation, *n* (%)	48 (48)	31 (54)	17 (40)	0.16
Mechanical ventilation, *n* (%)	61 (61)	42 (74)	19 (44)	< 0.01
NMBA, *n* (%)	14 (14)	6 (11)	8 (19)	0.26
Vasopressors, *n* (%)	74 (74)	46 (81)	28 (65)	0.11
Norepinephrine, μg/min	0.17 [0–0.75]	0.33 [0.07–101]	0.13 [0.0–0.40]	0.44
Dobutamine, μg/kg/min	3 [2.5–5]	0 [0–0]	0 [0–0]	0.88
MAP, mmHg	76 [67–86]	77 [59–86]	75 [66–86]	0.50
Heart rate, beats/min	96 [80–108]	98 [80–112]	95 [80–107]	0.09
Temperature, °C	37 [36.5–38.7]	36.9 [36.5–37.2]	37.0 [36.5–38.0]	0.23
FiO_2_	0.40 [0.30–0.50]	0.40 [0.30–0.50]	0.36 [0.28–0.40]	0.29
PEEP, cmH_2_O	5 [0–10]	6 [0–10]	0 [0–10]	0.05
PaO_2_, mmHg	79 [70–90]	78 [70–87]	80 [72–95]	0.45
PaCO_2_, mmHg	37 [32–43]	37 [33–44]	37 [32–43]	0.68
pH	7.40 [7.34–7.45]	7.37 [7.32–7.44]	7.43 [7.38–7.45]	0.39
ScvO_2_, %	69 [64–76]	67 [62–76]	70 [65–77]	0.40
Lactate, mEq/L	1.9 [1.3–2.7]	2 [1.3–3.1]	1.7 [1.2–2.4]	0.02
C-reactive protein, mg/L	260 [150–340]	260 [150–340]	260 [190–350]	0.63
Haemoglobin, mg/dl	9.5 [8.3–11.4]	10 [8.4–12]	9.6 [8.5–12]	0.63
Pro-ET-1, pg/ml	19 [14–36]	20 [15–48]	15 [8–31]	0.17
Mxa	0.29 [0.05–0.62]	0.47 [0.21–0.64]	0.23 [− 0.12–0.52]	< 0.01
Altered CAR, *n* (%)	50 (50)	34 (60)	27 (37)	0.04
Primary site of infection, *n* (%)				
Abdominal	46 (46)	30 (53)	16 (37)	0.16
Respiratory	28 (28)	14 (25)	14 (33)	0.50
Urinary tract	9 (9)	6 (11)	3 (7)	0.73
Soft tissue	8 (8)	2 (4)	6 (14)	0.07
Blood/CVC	4 (4)	3 (5)	1 (2)	0.63
Unknown	6 (6)	3 (5)	3 (7)	1
Pathogen^a^, *n* (%)				
GNB	45 (45)	23 (40)	22 (51)	0.32
GPB	35 (35)	23 (40)	12 (28)	0.21
Fungi	10 (10)	6 (11)	4 (9)	1
Virus/other	5 (5)	2 (4)	3 (7)	0.58
Unknown	23 (23)	13 (23)	10 (23)	1

*Abbreviations: APACHE II* Acute Physiology and Chronic Health Evaluation II, *CAR* Cerebral autoregulation, *CKD* Chronic kidney disease, *CVC* Central venous catheter, *FiO*_*2*_ Fraction of inspired oxygen, *GNB* Gram-negative bacteria, *GNP* Gram-positive bacteria, *ICU LOS* Intensive care unit length of stay, *MAP* Mean arterial pressure, *Mxa* Mean flow index, *NMBA* Neuromuscular blocking agent, *PaCO*_*2*_ Arterial carbon dioxide partial pressure, *PaO*_*2*_ Arterial oxygen partial pressure, *PEEP* Positive end-expiratory pressure, *ScvO*_*2*_ Central venous haemoglobin oxygen saturation

^a^ Total percentage can exceed 100 because of multiorganism infections

Data are expressed as count (%) or median [IQR]

### Cerebral autoregulation

**Fig. 1:**
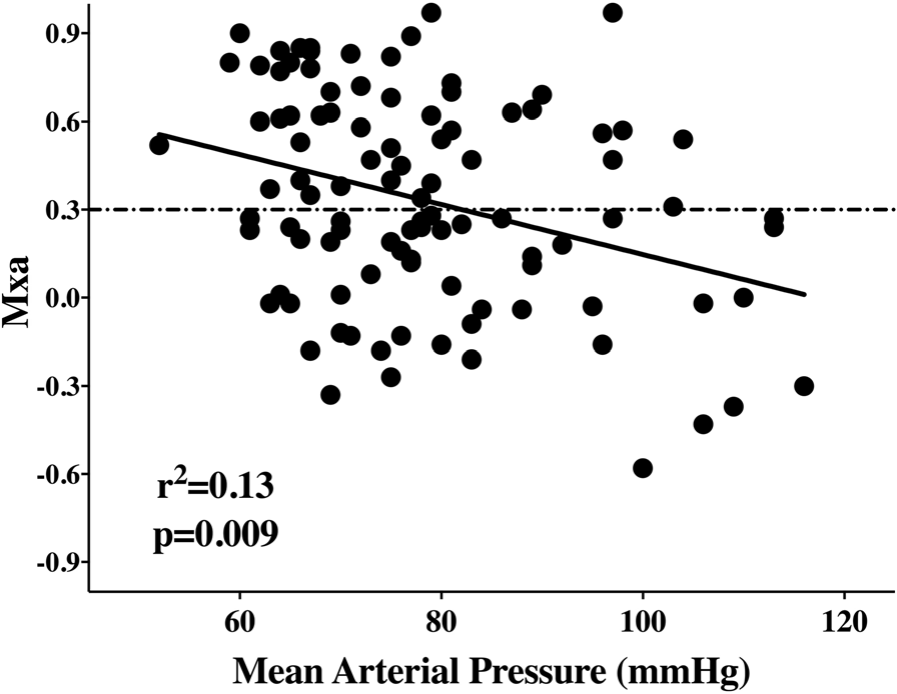
Correlation between mean flow index and mean arterial blood pressure

Mxa was 0.29 [0.05–0.62], and 50 (50%) patients had impaired CAR. The length of recording was 13 [10–18] minutes per patient. Mxa correlated with HR (*r* = 0.23; *p* = 0.02) and MAP (*r* = − 0.33; *p* < 0.01) (Additional file 1: Table S1). Mxa was higher in patients treated with NMBAs than in other patients (0.69 [0.19–0.84] *vs* 0.27 [0.03–0.57]; *p* = 0.03) (Additional file 1: Table S2). Mxa was also higher in patients with chronic kidney disease (CKD) or fungal infection than in the others (0.7 [0.25–0.85] vs 0.27 [0.01–0.59]; *p* = 0.02; and 0.69 [0.19–0.84] vs 0.27 [0.03–0.57]; *p* = 0.003, respectively). Mxa did not differ in patients who were receiving vasopressors compared with those who were not (0.38 [0.15–0.63] vs 0.22 [0.00–0.53]; *p* = 0.24), and no correlation between Mxa and dose of vasopressors was found. No association between Mxa and PaCO_2_ was found. In the multiple linear regression analysis, lower MAP, history of CKD, and fungal infection were independently associated with higher Mxa (Table 2, Fig. 1). Seventy-six patients were alive at ICU discharge, with no difference between centres (Additional file 1: Table S4). There was no difference in Mxa between survivors and non-survivors at ICU discharge (Mxa 0.27 [− 0.02–0.62] vs 0.43 [0.23–0.63]; *p* = 0.12).

**Table 2:** Multiple linear regression model to predict mean flow index

Independent variables	Unstandardized coefficients (95% CI)	Standardized coefficients	*p* Value
Mean arterial pressure (mmHg)	−0.007 (−0.012; −0.003)	−0.274	< 0.01
Heart rate (beats/min)	0.003 (−0.001; 0.006)	0.154	0.10
Chronic kidney disease	0.225 (0.007; 0.443)	0.188	0.04
Fungal infection	0.284 (0.069; 0.500)	0.166	0.01
NMBA	0.171 (−0.017; 0.360)	0.166	0.07

*NMBA* Neuromuscular blocking agent

### Sepsis-associated brain dysfunction

**Fig. 2:**
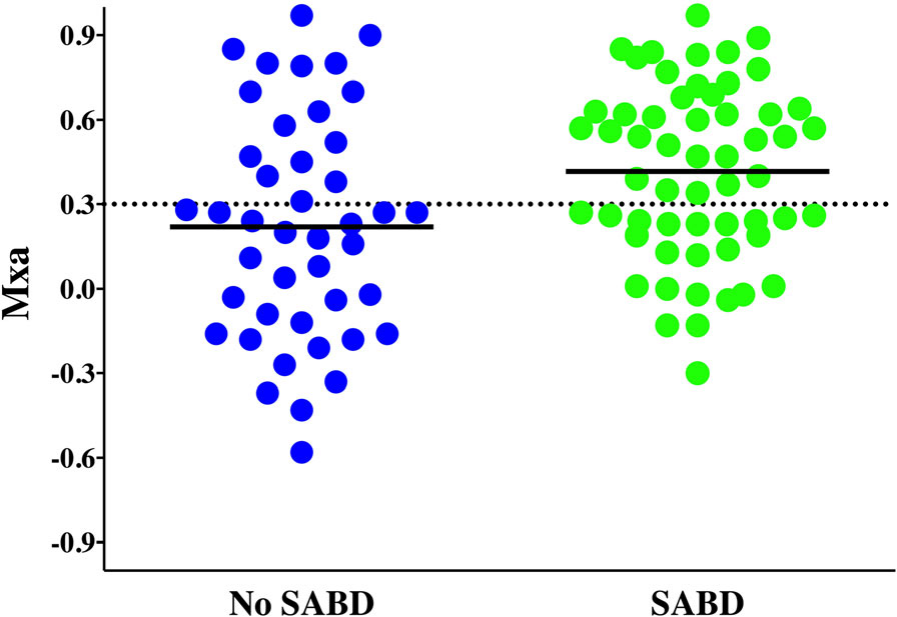
Mean flow index (Mxa) in patients with and without sepsis-associated encephalopathy (sepsis-associated brain dysfunction [SABD]). Mxa is higher in patients with SABD (0.37 ± 0.05 vs 0.18 ± 0.06; *p* = 0.03)

SABD was diagnosed in 57 patients. As expected, patients with SABD had higher APACHE II scores, longer ICU LOS and greater ICU mortality than patients without SABD (Table 1). These patients also had higher lactate levels (2.0 [1.3–3.1] vs 1.7 [1.2–2.4] mEq/L; *p* = 0.02) and were more likely to be on mechanical ventilation (42 of 57 [74%] vs 19 of 43 [44%]; *p* < 0.01) and to have a history of vascular disease (17 of 57 [30%] vs 3 of 43 [7%]; *p* < 0.01). SABD was more common in patients with altered CAR than in those with intact CAR (34 of 50 [68%] vs 23 of 50 [46%]; *p* = 0.04), and Mxa was higher in patients with SABD (0.47 [0.21–0.64] vs 0.23 [− 0.12–0.52]; *p* < 0.01) (Table 1, Fig. 2). Results were similar when the six patients in whom neurological evaluation was not possible were excluded from the analysis (0.58 [0.25–0.68] vs 0.23 [− 0.12–0.52]; *p* < 0.01). In multivariable analysis, higher Mxa, vascular disease and mechanical ventilation were independent predictors of SABD (Table 3). The logistic regression model correctly classified 74% of patients. The AUC for Mxa to predict SABD was 0.65 (95% CI 0.53–0.76; *p* < 0.01). The best Mxa cut-off to predict SABD was 0.18 (sensitivity 79%, specificity 47%).

**Table 3:** Binary logistic regression to predict sepsis-associated brain dysfunction

Independent variables	OR (95% CI)	*p* Value
Vascular disease	0.22 (0.05; 0.88)	0.03*
Mechanical ventilation	0.12 (0.02; 0.88)	0.04*
Mxa	5.11 (1.40;18.65)	0.02*
Sedation	1.14 (0.30; 4.39)	0.85
Serum lactate (mEq/L)	1.32 (− 0.98; 1.77)	0.07
PEEP (mmHg)	0.90 (0.75; 1.09)	0.30

*Mxa* Mean flow index, *PEEP* Positive end-expiratory pressure

## Discussion

In this large, multicentre study, CAR was altered in half of patients with sepsis and was independently associated with the occurrence of SABD, although not with ICU survival. Lower MAP, a history of CKD, and fungal infection were associated with altered CAR. This study represents the largest cohort of patients affected by sepsis in whom CAR has been tested and its relationship with SABD analysed considering potential confounders. The present findings thus support the concept that cerebral hypoxia could contribute to the development of SABD.

A recent study identified several risk factors for the occurrence of SABD (i.e., acute renal failure, dysglycaemia, hypercapnia, hypernatremia and *Staphylococcus aureus* infection), but no systemic hemodynamic variables were identified [19]. Two previous studies evaluating static CAR (i.e., changes in CBF following the manipulation of ABP under steady state using vasopressors) reported preserved CAR during sepsis [14, 20], whereas another reported impaired CAR in patients with septic shock [15]. In contrast, several studies assessing dynamic CAR (i.e., changes in CBF in response to sudden spontaneous or induced [head-up tilt, Valsalva manoeuvre or standing up]) fluctuations of ABP showed that impaired CAR was more frequently observed in patients with sepsis than in those without it [12] or in patients with sepsis with SABD when than in those without brain dysfunction [11, 13]. It has previously been suggested that dynamic CAR may be more easily impaired than static CAR in different diseases because of the different control mechanisms involved [21]. One potential explanation would be the dysregulation of the autonomic nervous system or of the baroreceptor reflex arc, which preferentially influences the dynamic pathway, that is typically observed in sepsis [22]. The poor temporal resolution of static CAR assessment could not adequately describe the changes in CBF that occur during sepsis, where significant hemodynamic fluctuations, particularly in the early stage of the disease, are reported.

In our study, SABD was related to well-known risk factors for brain dysfunction, such as the presence of vascular disease (i.e., increased risk of cerebrovascular injury) and the use of mechanical ventilation (i.e., increased risk of delirium) [23]. Our findings also suggest that impairment of dynamic CAR has a significant influence on the occurrence of SABD. Given the increased levels of energetic substrates required by complex brain activity, even a slight reduction in CBF, as has been observed in patients with sepsis [24], may impair superior cognitive functions. Alteration in CAR together with microcirculatory alterations could explain local hypoperfusion in absence of severe systemic hypotension. Several potential causes of impaired CAR during sepsis have been identified, including nitric oxide accumulation, blood-brain barrier breakdown due to neuro-inflammation, and impaired microcirculation [9, 25, 26]. This is the first study, to the best of our knowledge, where the association of impaired CAR with the occurrence of SABD has been evaluated in a large cohort with correction for several confounders, and the present results strongly support the concept that cerebral hypoxia could contribute to the development of SABD.

Renal and cerebral vasculature share multiple characteristics, such as high blood flow rates, high pressure load and effective autoregulation of local flow. Chronic renal disease negatively affects microvascular cerebral function, favouring endothelial dysfunction, chronic inflammation, accumulation of urea or of vasoactive species, and alterations in sympathetic nervous system-driven vascular resistance [27]. CAR has been found to be altered in paediatric patients with chronic renal dysfunction or in adult critically ill patients with acute kidney injury requiring renal replacement therapy [28, 29]. The association between CKD and altered CAR in our study supports the hypothesis of kidney-brain crosstalk and negative effects of renal dysfunction on cerebral vasculature. The association between fungal infection and altered CAR is not easily explained. Specific effects of non-cerebral fungal infections on brain endothelial and microvascular function are unknown. Fungal infections are highly invasive, especially in cases of abdominal or respiratory infection [30]; however, only ten patients in our cohort had fungal infections, and the possibility that a subclinical fungal involvement of the cerebral parenchyma would be the main reason for impaired CAR in this cohort remains unlikely.

Although a lower baseline MAP was a significant predictor of impaired CAR, we did not specifically assess the lower limit (LL) of CAR integrity, which may significantly vary in healthy subjects and can be affected by different chronic conditions; that is, in patients with chronic hypertension, the LL will be shifted towards higher values than in normotensive patients, and CBF could become “pressure-dependent” for lower values of MAP than in physiologic conditions [31]. In one experimental study on rats, LL was also right-shifted, which would translate into a higher risk of cerebral hypoperfusion for MAP values considered as “normal” in clinical practice [32]. Whether higher blood pressures should be targeted in selected patients with sepsis to avoid cerebral hypoperfusion remains to be further evaluated. Interestingly, although several studies reported a potential role of hypercapnia in the CAR impairment during sepsis [15, 20], no correlation was found between PaCO_2_ and autoregulation in our cohort. Thus, in our cohort CAR is impaired, regardless of the PaCO_2_ level. In a recent meta-analysis, there was no clear association between PaCO_2_ and/or CO_2_ reactivity with impaired CAR during sepsis [33]. As such, future studies prospectively evaluating the effects of changes in CO_2_ and CAR function are warranted.

In contrast to the previous study by Bindra et al. [34], we observed no association between altered CAR and mortality. However, their study differed substantially with regard to patient characteristics (i.e., 28 patients in septic shock, all sedated and on mechanical ventilation), method of CAR assessment (i.e., use of near-infrared spectroscopy and not TCD to evaluate CAR) and timing of outcome assessment (i.e., 3 months) compared with our cohort. Furthermore, brain dysfunction in patients with sepsis is not a direct cause of death in the early phase, but it is associated with long-term cognitive alterations, which were not assessed in this study.

Our study has several strengths. To date, it represents the largest cohort of patients with sepsis in whom CAR has been tested. We included patients affected by sepsis of different severities; risk of bias in selection and treatment is limited by the multicentre nature of the study. We assessed CAR using Mxa, a validated method which enables CAR assessment using spontaneous fluctuations in MAP, thus limiting potentially stressful procedural and/or pharmacological interventions. Such fluctuations depend on specific neuronal pathways and occur in healthy or critically ill subjects at very low frequency or as a physiologic response to variations in intra-thoracic pressures [35]. Mxa has been validated in patients with traumatic brain injury [36] and is already used in patients with sepsis [13].

The present study also has several limitations. First, TCD technique only evaluates CBF in large intracranial arteries, thus impairment of microcirculation potentially leading to local ischaemia could not be specifically analysed. Second, Mxa is a mathematical simplification of a complex biological phenomenon and only investigates dynamic components of CAR. Static and dynamic CAR have been reported to be affected differently in healthy volunteers and patients with sepsis [20]. Moreover, Mxa is only one method used to assess CAR, and different findings might have been obtained if other methods had been used [37]. The threshold traditionally applied to define altered autoregulation is Mxa > 0.3; however, the absolute value of such thresholds has been questioned, and Mxa should be considered a continuous index that reflects a wide spectrum of severity of CAR impairment. Third, the diagnosis of SABD was based on Glasgow Coma Scale score and some signs of delirium and was not standardized on specific scales or scores, and no additional findings from brain monitoring were used. Nevertheless, we lacked widely accepted criteria to define septic encephalopathy [2]. The Confusion Assessment Method for the ICU has been validated for diagnosis of delirium, but its benefit in SABD diagnosis has been questioned [38]. In particular, the spectrum of clinical presentation of SABD is much wider than delirium alone and includes behavioural and personality changes and even post-traumatic stress disorder symptoms, so that unstructured assessment may be a better screen for this condition [39]. Also, none of the available neuromonitoring tools (i.e., electroencephalography, biomarkers or neuroimaging) are specific for SABD [40]. Moreover, our clinical definition was very similar to the definition used in other recent studies dealing with SABD [19]. Fourth, sedation generates different effects in the autonomic nervous system and CAR [41]. However, only half of the patients received sedatives and/or analgesics with different classes of drugs, so specific subgroup analyses would have been limited. Fifth, we assessed CAR only once in the early phase of sepsis, whereas this might improve over time in the first days following sepsis diagnosis [13]. However, in this study, only CAR assessed on day 1 correlated with the occurrence of SABD thereafter, suggesting that early alterations have the largest influence on the development of brain dysfunction in these patients.

## Conclusions

In this large cohort of patients with sepsis, CAR assessed by Mxa was altered in half of the cases. Altered CAR was found to be independently associated with septic encephalopathy, but not with survival at ICU discharge.

## Additional file

Additional file 1: **Table S1.** Association of continuous variables with Mxa. Pearson’s correlation and Spearman’s rank-order correlation were used for normally distributed and non-normally distributed variables, respectively. **Table S2.** Association of categorical variables with Mxa. **Table S3.** Comparison between patients enrolled in Belgium and Spain. Data are expressed as count (%) or median [IQR]. **Table S4.** Comparison between survivors and non-survivors. (DOCX 39 kb)

## Abbreviations

ABP: Arterial blood pressure
APACHE II: Acute Physiology and Chronic Health Evaluation II
CAR: Cerebral autoregulation
CBF: Cerebral blood flow
CKD: Chronic kidney disease
CPP: Cerebral perfusion pressure
CVC: Central venous catheter
FiO_2_: Fraction of inspired oxygen
FV: Flow velocity
GNB: Gram-negative bacteria
GNP: Gram-positive bacteria
HR: Heart rate
ICU: Intensive care unit
LL: Lower limit
LOS: Length of stay
MAP: Mean arterial pressure
Mxa: Mean flow index
NMBA: Neuromuscular blocking agent
PaCO_2_: Arterial carbon dioxide partial pressure
PaO_2_: Arterial oxygen partial pressure
PEEP: Positive end-expiratory pressure
SABD: Sepsis-associated brain dysfunction
ScvO_2_: Central venous haemoglobin oxygen saturation
TCD: Transcranial Doppler
