# Supplementary material for: Impaired cerebral autoregulation is associated with brain dysfunction in patients with sepsis
Source: Crit Care. 2018 Dec 4;22:327. doi: 10.1186/s13054-018-2258-8 (PMC6280405; doi:10.1186/s13054-018-2258-8)
Supplement: Supplementary file 1 — Table S1. Association of continuous variables with Mxa. Pearson’s correlation and Spearman’s rank-order correlation were used for normally distributed and non-normally distributed variables, respectively. Table S2. Association of categorical variables with Mxa. Table S3. Comparison between patients enrolled in Belgium and Spain. Data are expressed as count (%) or median [IQR]. Table S4. Comparison between survivors and non-survivors. (DOCX 39 kb) [file 13054_2018_2258_MOESM1_ESM.docx]

**Table S1.** Association of continuous variables with Mxa. Pearson’s correlation and Spearman’s rank-order correlation were used for normally distributed and non-normally distributed variables, respectively.

| **Mxa vs:** | **Correlation Coefficient**  **(*r*/*r_s_*)** | **p-Value** |
| --- | --- | --- |
| Age, years | -0.01 | 0.90 |
| APACHE II score on admission | 0.06 | 0.57 |
| ICU LOS, days | 0.06 | 0.59 |
| Norepinephrine (mcg/min) | 0.16 | 0.1 |
| Dobutamine (mcg/Kg/min) | -0.10 | 0.36 |
| MAP, mmHg | -0.33 | <0.01 |
| HR, bpm | 0.23 | 0.02 |
| Temperature, °C | 0.02 | 0.82 |
| FiO_2_ | 0.30 | <0.01 |
| PEEP, cmH_2_O | 0.04 | 0.69 |
| PaO_2_, mmHg | -0.03 | 0.79 |
| PaCO_2_, mmHg | 0.12 | 0.23 |
| pH | -0.18 | 0.07 |
| ScvO_2_, % | -0.10 | 0.35 |
| Lactate, mEq/L | 0.12 | 0.24 |
| CRP, mg/L | 0.04 | 0.67 |
| Hemoglobin, mg/dL | -0.08 | 0.41 |
| Pro-ET-1, pg/L | -0.12 | 0.53 |

APACHE II Acute Physiology and Chronic Health Evaluation II score; ICU LOS intensive care unit length of stay; HR heart rate; MAP Mean Arterial Pressure; FiO_2_ fraction of inspired oxygen; PEEP positive end-expiratory pressure; PaO_2_ arterial oxygen partial pressure; PaCO_2_ arterial carbon dioxide partial pressure; ScvO_2_ central venous hemoglobin oxygen saturation; pro-ET-1 pro-endothelin-1; CRP C-reactive protein

**Table S2.** Association of categorical variables with Mxa.

| **Mxa** | | **p-Value** |
| --- | --- | --- |
| **Male (n=72)** | **Female (n=28)** |  |
| 0.36 [0.11 - 0.63] | 0.24 [0.01 - 0.61] | 0.40 |
| **SABD (n=57)** | **No SABD (n=43)** |  |
| 0.47 [0.21 – 0.68] | 0.27 [0.08 – 0.63] | <0.01 |
| **Survivors (n=76)** | **Non survivors (n=24)** |  |
| 0.27 [-0.02 - 0.62] | 0.43 [0.23 - 0.63] | 0.12 |
| **CKD (n=10)** | **Normal baseline renal function (n=90)** |  |
| 0.7 [0.25-0.85] | 0.27 [0.01-0.59] | 0.02 |
| **Vascular disease (n=20)** | **No vascular disease (n=80)** |  |
| 0.47 [0.14-0.63] | 0.27 [0.02-0.62] | 0.40 |
| **Diabetes mellitus (n=23)** | **No diabetes mellitus (n=77)** |  |
| 0.50 [0.22-0.63] | 0.27 [0.00-0.62] | 0.16 |
| **Smoking (n=19)** | **No smoking (n=81)** |  |
| 0.31 [-0.12-0.63] | 0.28 [0.12-0.62] | 0.35 |
| **Arterial hypertension (n=45)** | **Normal baseline arterial pressure (n=55)** |  |
| 0.36 [0.01-0.64] | 0.27 [0.06-0.54] | 0.57 |
| **Sedation (n=48)** | **No sedation (n=52)** |  |
| 0.33 [0.00-0.67] | 0.28 [0.12-0.59] | 0.98 |
| **Mechanical ventilation (n=61)** | **Spontaneous breathing (n=39)** |  |
| 0.35 [-0.01-0.63] | 0.27 [0.13-0.62] | 0.87 |
| **NMBA (n=14)** | **No NMBA (n=86)** |  |
| 0.69 [0.19-0.84] | 0.27 [0.03-0.57] | 0.03 |
| **Vasopressors (n=74)** | **No vasopressors (n=26)** |  |
| 0.38 [0.15-0.63] | 0.22 [0.00-0.53] | 0.24 |
| **Abdominal infection (n=46)** | **Non-abdominal infection (n=64)** |  |
| 0.43 [0.16-0.65] | 0.27 [-0.02-0.57] | 0.10 |
| **Respiratory infection (n=28)** | **Non-respiratory infection (n=72)** |  |
| 0.26 [-0.13-0.56] | 0.39 [0.13-0.63] | 0.14 |
| **Urinary tract infection (n=9)** | **Non-urinary tract infection (n=91)** |  |
| 0.24 [-0.06-0.52] | 0.31 [0.11-0.63] | 0.34 |
| **Soft tissue infection (n=8)** | **Non-soft tissue infection (n=92)** |  |
| 0.39 [-0.09-0.49] | 0.28 [0.05-0.63] | 0.70 |
| **Blood/CVC infection (n=4)** | **No-blood/CVC infection (n=96)** |  |
| 0.43 [0.04-0.77] | 0.30 [0.05-0.62] | 0.66 |
| **Unknown site of infection (n=6)** | **Known site of infection (n=94)** |  |
| 0.40 [-0.11-0.83] | 0.30 [0.07-0.62] | 0.79 |
| **GNB infection (n=45)** | **No-GNB infection (n=55)** |  |
| 0.28 [0.05-0.59] | 0.31 [0.04-0.68] | 0.40 |
| **GPB infection (n=35)** | **No-GPB infection (n=65)** |  |
| 0.47 [0.00-0.63] | 0.27 [0.10-0.61] | 0.46 |
| **Fungal infection (n=10)** | **No-fungal infection (n=90)** |  |
| 0.63 [0.55-0.91] | 0.27 [0.00-0.58] | <0.01 |
| **Viral/Other infection (n=5)** | **Bacterial/fungal/unknown pathogen (n=95)** |  |
| 0.31 [0.27-0.55] | 0.28 [0.01-0.62] | 0.53 |
| **Unknown pathogen (n=23)** | **Known pathogen infection (n=77)** |  |
| 0.26 [0.04-0.57] | 0.35 [0.05-0.63] | 0.66 |

Data are expressed as median [IQR]. SAE sepsis-associated encephalopathy; CKD chronic kidney disease; NMBA neuromuscular blocking agent; GNB Gram-negative bacteria; GNP Gram-positive bacteria; CVC central venous catheter; SABD = sepsis-associated brain dysfunction

**Table S3**. Comparison of patient characteristics according to site of enrolment.

|  | **Belgian cohort (n=72)** | **Spanish cohort (n=28)** | **p-value** |
| --- | --- | --- | --- |
| Age (years) | 62 [50 - 68] | 71 [57 - 77] | 0.03 |
| Male, n (%) | 51 (71) | 25 (89) | 0.07 |
| APACHE II score | 21 [15 - 27] | 21 [14 - 26] | 0.79 |
| ICU LOS (days) | 7 [4 - 13] | 11 [5 - 13] | 0.71 |
| SAE, n (%) | 21 (57) | 16 (57) | 1.00 |
| Alive at ICU discharge, n (%) | 56 (78) | 20 (71) | 0.60 |
| Intact cerebral autoregulation, n (%) | 32 (44) | 18 (64) | 0.12 |
| **COMORBIDITIES** | | | |
| CKD, n (%) | 9 (13) | 1 (4) | 0.28 |
| Vascular disease (any district), n (%) | 14 (19) | 6 (21) | 0.79 |
| Diabetes mellitus, n (%) | 17 (24) | 5 (18) | 0.60 |
| Smoking, n (%) | 12 (17) | 7 (25) | 0.40 |
| Arterial hypertension, n (%) | 29 (40) | 15 (54) | 0.27 |
| **AT TIME OF ASSESSMENT OF CEREBRAL AUTOREGULATION** | | | |
| Sedation, n (%) | 25 (35) | 23 (82) | <0.01 |
| Mechanical ventilation, n (%) | 37 (51) | 24 (86) | <0.01 |
| NMBA, n (%) | 10 (14) | 4 (14) | 1.00 |
| Vasopressors, n (%) | 52 (72) | 22 (79) | 0.62 |
| Norepinephrine, mcg/min | 0.17 [0 - 1.0] | 0.15 [0.1 - 0.5] | 0.01 |
| Dobutamine, mcg/Kg/min | 0 [0 - 0] | 0 [0 - 1] | 0.94 |
| MAP, mmHg | 71 [66 - 79] | 90 [80 - 102] | <0.01 |
| Heart rate, bpm | 100 [84 - 108] | 89 [77 - 112] | 0.41 |
| Temperature, °C | 37 [36 - 38] | 37 [37 - 38] | 0.63 |
| FiO_2_ | 0.4 [0.3 - 0.5] | 0.4 [0.3 - 0.5] | 0.70 |
| PEEP, cmH_2_O | 5 [0 - 10] | 8 [5 - 10] | 0.07 |
| PaO_2_, mmHg | 78 [69 - 91] | 80 [73 - 86] | 0.39 |
| PaCO_2_, mmHg | 40 [33 - 45] | 40 [33 - 45] | 0.19 |
| pH | 7.40 [7.34 - 7.45] | 7.39 [7.34 - 7.44] | 0.54 |
| ScvO_2_, % | 67 [62 - 74] | 74 [67 - 79] | 0.05 |
| Lactate, mEq/L | 2 [1.5 - 2.8] | 1.8 [1.2 - 2.6] | 0.79 |
| C-reactive protein, mg/L | 265 [143 - 340] | 251 [193 - 356] | 0.68 |
| Hemoglobin, mg/dL | 9.5 [8.3 - 11.4] | 11 [9 - 12.8] | <0.01 |
| Mxa | 0.38 [0.18 - 0.64] | 0.19 [-0.18 - 0.56] | <0.01 |
| Primary site of infection, n (%):   - *Abdominal* - *Respiratory* - *Urinary tract* - *Soft tissue* - *Blood/CVC* - *Unknown* | 37 (51)  13 (18)  7 (10)  8 (11)  2 (3)  6 (8) | 9 (32)  15 (54)  2 (7)  0 (0)  2 (7)  0 (0) | 0.12  <0.01  1.00  0.10  0.31  0.18 |
| Pathogen*^a^*, n (%):   - *GNB* - *GPB* - *Fungi* - *Virus/Other* - *Unknown* | 34 (47)  24 (33)  10 (14)  2 (3)  17 (24) | 11 (39)  11 (39)  0 (0)  3 (11)  6 (21) | 0.51  0.64  0.06  0.13  1.00 |

Data are expressed as counts (%) or median [IQR]. SAE sepsis-associated encephalopathy; APACHE II Acute Physiology and Chronic Health Evaluation II score; ICU LOS intensive care unit length of stay; CKD chronic kidney disease; NMBA neuromuscular blocking agent; MAP mean arterial pressure; FiO_2_ fraction of inspired oxygen; PEEP positive end-expiratory pressure; PaO_2_ arterial oxygen partial pressure; PaCO_2_ arterial carbon dioxide partial pressure; ScvO_2_ central venous hemoglobin oxygen saturation; Mxa mean flow index; CVC central venous catheter; GNB Gram-negative bacteria; GNP Gram-positive bacteria . *^a^* total % can exceed 100 because of multi-organism infections.

**Table S4.** Comparison between survivor and non-survivors.

|  | **Survivors**  **(n=76)** | **Non Survivors (n=24)** | **p-value** |
| --- | --- | --- | --- |
| Age (years) | 62 [52 - 70] | 67 [52 - 75] | 0.39 |
| Male, n (%) | 59 (78) | 17 (71) | 0.58 |
| APACHE II score | 21 [14 - 26] | 20 [15 - 28] | 0.17 |
| ICU LOS (days) | 8 [4 - 13] | 5 [3 - 10] | 0.47 |
| SAE, n (%) | 36 (47) | 21 (88) | <0.01 |
| Alive at ICU discharge, n (%) | 56 (78) | 20 (71) | 0.60 |
| Intact cerebral autoregulation, n (%) | 40 (53) | 10 (42) | 0.48 |
| **COMORBIDITIES** | | | |
| CKD, n (%) | 8 (11) | 2 (8) | 1.00 |
| Vascular disease (any district), n (%) | 13 (17) | 7 (29) | 0.24 |
| Diabetes mellitus, n (%) | 17 (22) | 5 (21) | 1.00 |
| Smoking, n (%) | 14 (18) | 5 (21) | 0.77 |
| Arterial hypertension, n (%) | 33 (43) | 11 (46) | 1.00 |
| **AT TIME OF ASSESSMENT OF CEREBRAL AUTOREGULATION** | | | |
| Sedation, n (%) | 33 (43) | 15 (63) | 0.16 |
| Mechanical ventilation, n (%) | 40 (53) | 21 (88) | 0.03 |
| NMBA, n (%) | 8 (11) | 6 (25) | 0.09 |
| Vasopressors, n (%) | 52 (68) | 22 (79) | 0.03 |
| Norepinephrine, mcg/min | 0.14 [0 - 0.5] | 0.57 [0.15 - 1.03] | 0.53 |
| Dobutamine, mcg/Kg/min | 0 [0 - 0] | 0 [0 - 1.12] | 0.12 |
| MAP, mmHg | 75 [67 - 86] | 79 [71 - 86] | 0.38 |
| Heart rate, bpm | 94 [78 - 107] | 100 [89 - 120] | 0.07 |
| Temperature, °C | 37 [36.6 - 37.8] | 37 [36.1 - 37.7] | 0.63 |
| FiO_2_ | 0.4 [0.3 - 0.4] | 0.5 [0.3 - 0.6] | 0.70 |
| PEEP, cmH_2_O | 5 [0 - 10] | 8 [5 - 10] | 0.02 |
| PaO_2_, mmHg | 80 [70 - 90] | 76 [69 - 98] | 0.05 |
| PaCO_2_, mmHg | 37 [32 - 43] | 39 [32 - 45] | 0.63 |
| pH | 7.41 [7.36 - 7.45] | 7.34 [7.29 - 7.40] | 0.33 |
| ScvO_2_, % | 68 [65 - 75] | 73 [59 - 79] | 0.71 |
| Lactate, mEq/L | 1.7 [1.2 - 2.6] | 1.9 [2.5 - 5.4] | 0.02 |
| C-reactive protein, mg/L | 289 [190 - 350 | 235 [95 - 307] | 0.19 |
| Hemoglobin, mg/dL | 9.8 [8.4 - 12] | 9.9 [8.4 - 11.9] | 0.85 |
| Pro-ET-1, pg/L | 0.18 [0.13 - 0.34] (n=26) | 0.35 [0.20 - na]  (n=3) | 0.58 |
| Mxa | 0.27 [-0.02 - 0.62] | 0.43 [0.24 - 0.63] | 0.12 |
| Primary site of infection, n (%):   - *Abdominal* - *Respiratory* - *Urinary tract* - *Soft tissue* - *Blood/CVC* - *Unknown* | 35 (46)  19 (25)  8 (11)  7 (9)  3 (4)  4 (5) | 11 (46)  9 (38)  1 (4)  1 (4)  1 (4)  2 (8) | 1.00  0.30  0.68  0.68  1.00  0.63 |
| Pathogen*^a^*, n (%):   - *GNB* - *GPB* - *Fungi* - *Virus/Other* - *Unknown* | 35 (46)  25 (33)  8 (11)  4 (5)  17 (22) | 10 (42)  10 (42)  2 (8)  1 (4)  6 (25) | 0.81  0.48  1.00  1.00  0.79 |

Data are expressed as counts (%) or median [IQR]. SAE sepsis-associated encephalopathy; APACHE II Acute Physiology and Chronic Health Evaluation II score; ICU LOS intensive care unit length of stay; CKD chronic kidney disease; NMBA neuromuscular blocking agent; map mean arterial pressure; FiO_2_ fraction of inspired oxygen; PEEP Positive End Expiratory Pressure; PaO_2_ arterial oxygen partial pressure; PaCO_2_ arterial carbon dioxide partial pressure; ScvO_2_ central venous hemoglobin oxygen saturation; pro-ET-1 pro-Endothelin-1; na not available; Mxa mean flow index; CVC central venous catheter; GNB Gram-negative bacteria; GNP Gram-positive bacteria . *^a^* total % can exceed 100 because of multi-organism infections.
